# Supplementary material for: MIND bomb 2 prevents RIPK1 kinase activity-dependent and -independent apoptosis through ubiquitylation of cFLIPL
Source: Commun Biol. 2021 Jan 19;4:80. doi: 10.1038/s42003-020-01603-y (PMC7815719; doi:10.1038/s42003-020-01603-y)
Supplement: Supplementary file 2 — Supplementary Information [file 42003_2020_1603_MOESM2_ESM.pdf]

MIND bomb 2 prevents RIPK1 kinase activity-dependent and -independent apoptosis through ubiquitylation of $cFLIP_L$

Osamu Nakabayashi, Hirotaka Takahashi, Kenta Moriwaki, Sachiko Komazawa-Sakon, Fumiaki Ohtake, Shin Murai, Yuichi Tsuchiya, Yuki Koyahara, Yasushi Saeki, Yukiko Yoshida, Soh Yamazaki, Fuminori Tokunaga, Tatsuya Sawasaki, Hiroyasu Nakano

Supplementary Materials

Supplementary Figures 1~20

Supplementary Data Files 1~8

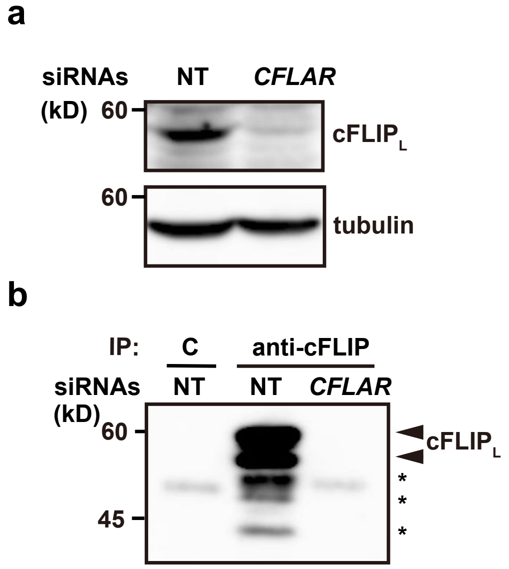

Supplementary Fig. 1 Immunoprecipitates with anti-cFLIP antibody exhibit multiple bands of $cFLIP_L$. WT HeLa cells were transfected with non-target (NT) or *CFLAR* siRNAs. Knockdown of $cFLIP_L$ by *CFLAR* siRNA was verified by immunoblotting (**a**). After transfection, cells were immunoprecipitated with control IgGs (C) or anti-cFLIP antibody, and the immunoprecipitates were analyzed by anti-cFLIP antibody (**b**). The upper and lower arrowheads indicate modified and unmodified $cFLIP_L$, respectively. Asterisks indicate degraded bands of $cFLIP_L$. Results are representative of two independent experiments.

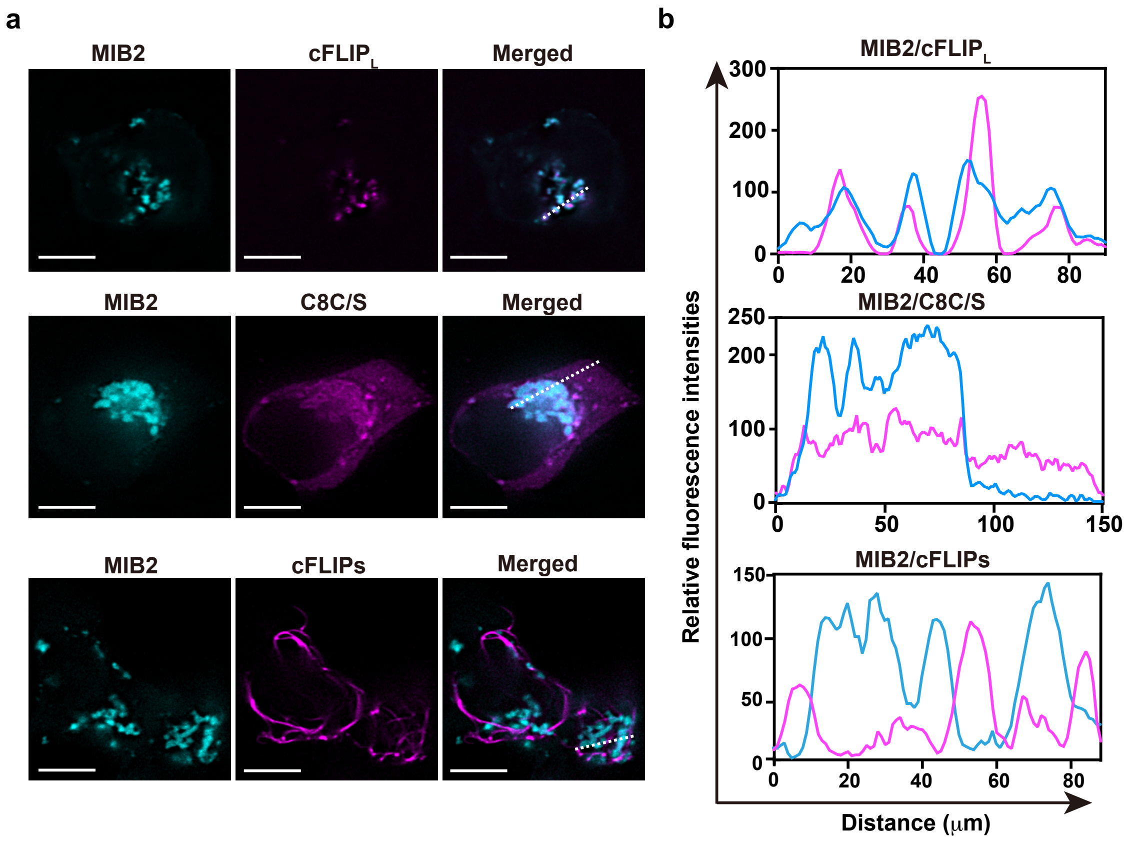

Supplementary Fig. 2 MIB2 colocalizes with cFLIP$_L$, but not cFLIPs or caspase 8. **a, b** HEK293 cells were co-transfected with GFP-MIB2 along with DsRed-cFLIP$_L$, DsRed-cFLIPs, or DsRed-C8C/S. Cellular localization of the indicated proteins was analyzed by DeltaVision (**a**). C8C/S indicates a protease-inactive mutant of caspase 8. Relative fluorescence intensities on the dotted white lines (merged images) were analyzed by Image J and are expressed as cyan (MIB2) and magenta lines (cFLIP$_L$, C8C/S, and cFLIPs), respectively (**b**). At least 5 cell images per transfection were collected. Scale bar, 10 μm. Results are representative of two or three independent experiments.

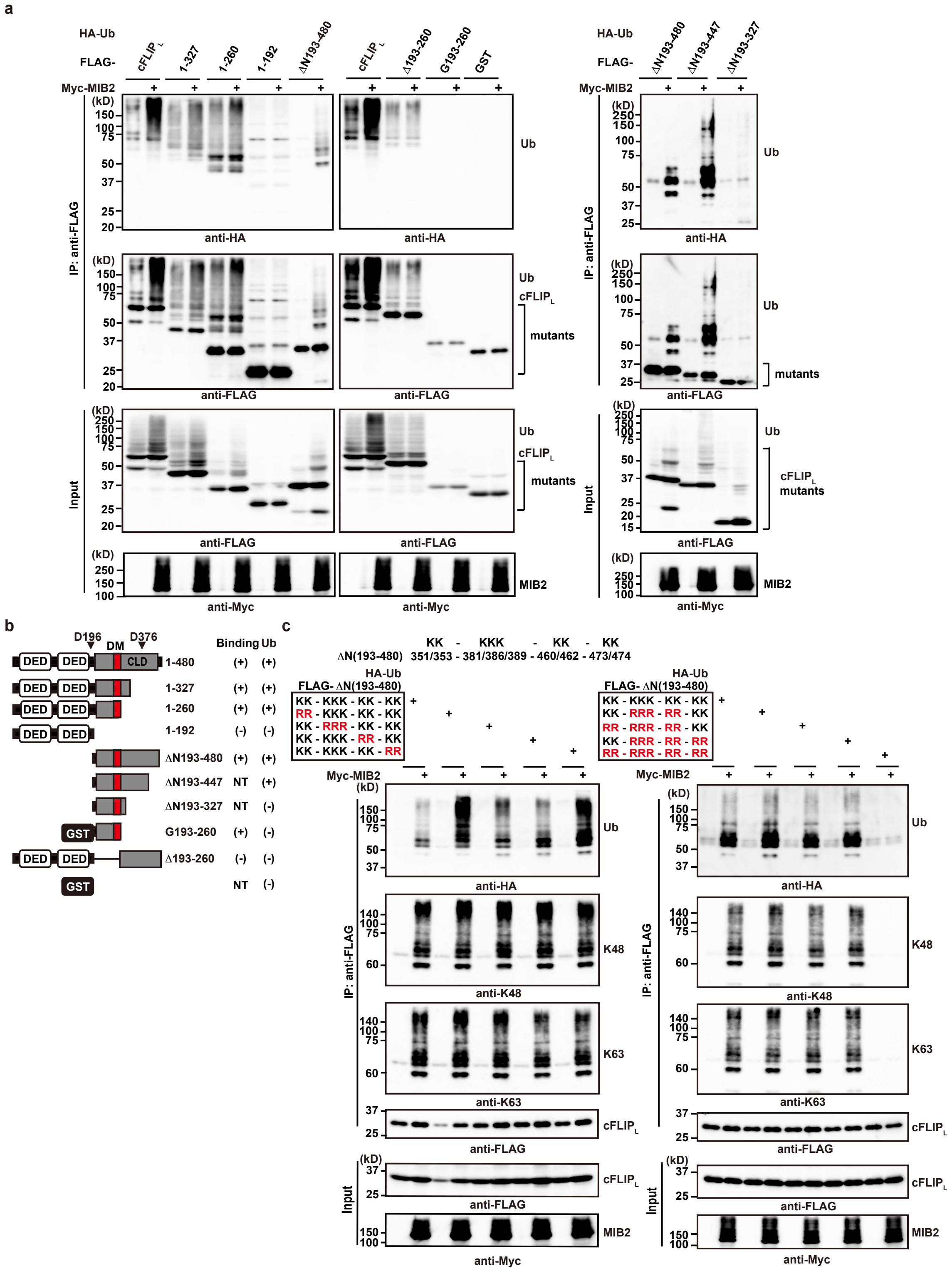

Supplementary Fig. 3 MIB2 ubiquitylates multiple sites of cFLIP$_L$. a HEK293 cells were transfected with cFLIP$_L$ or deletion mutants of cFLIP$_L$ containing indicated amino acids along with or without MIB2 and HA-Ub. At 24 hours after transfection, cell lysates were immunoprecipitated with anti-FLAG antibody, ubiquitylation of cFLIP$_L$

and its mutants was analyzed by immunoblotting with the indicated antibodies. Protein expression was verified by immunoblotting. **b** Diagrams of $cFLIP_L$ and its deletion mutants, and their binding to MIB2 and ubiquitylation. A deletion mutant containing the indicated amino acids was expressed as GST fusion protein with N-terminal FLAG tag (G193-260), since small polypeptides might be easily degraded in the cells. DED, death effector domain; DM, a docking site for MIB2; CLD, caspase-like domain; GST, glutathione S-transferase. D196 and D376 indicate aspartic acids cleaved by activated caspase 8. **c** MIB2 ubiquitylates multiple lysines in the C-terminal portion of $cFLIP_L$. ΔN193-480 and its lysine to arginine (KR) mutants at the indicated positions were transfected and analyzed as in **a**. Each number indicates lysine residues' positions, and lysines replaced with arginines are highlighted with red characters. MT indicates the mutants of ΔN193-480. Results are representative of two or three independent experiments.

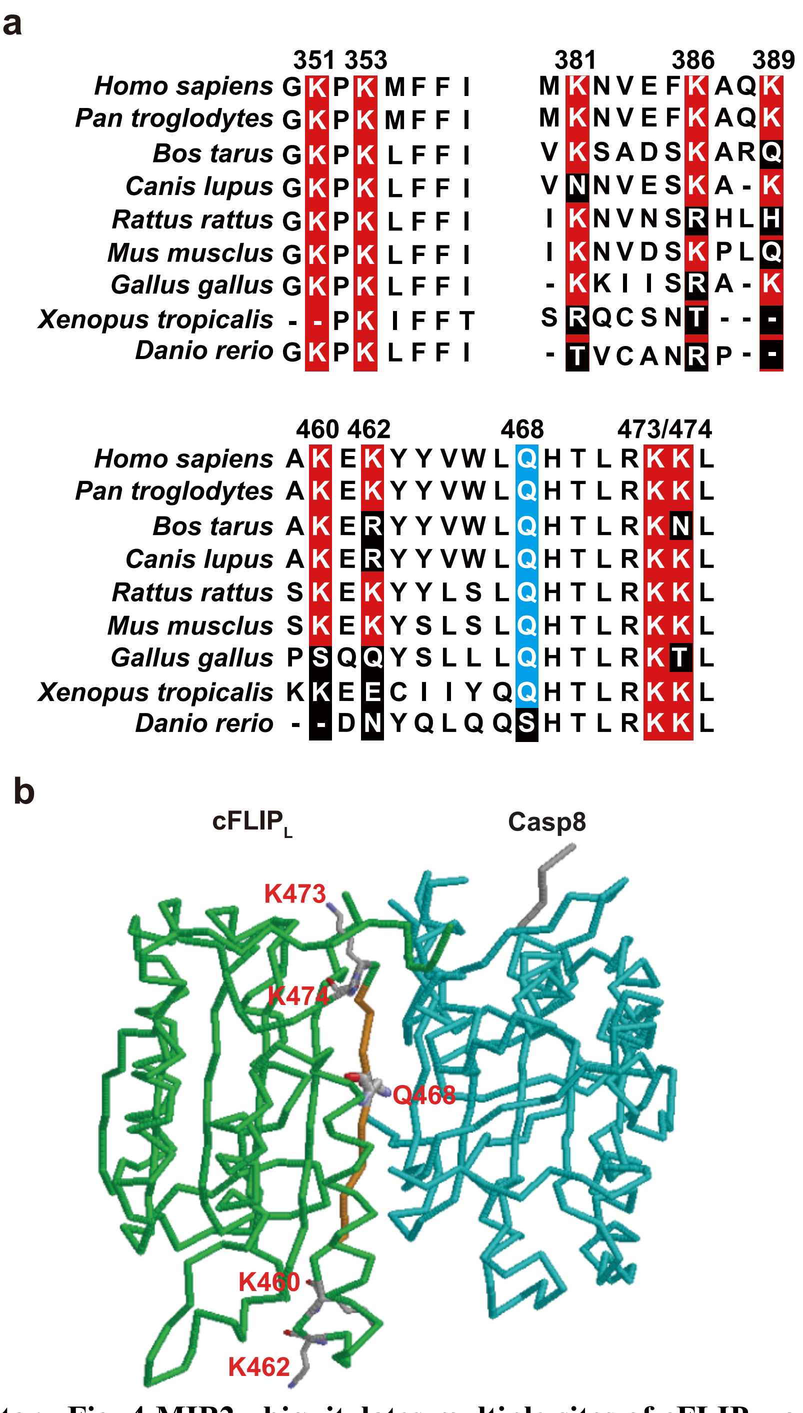

Supplementary Fig. 4 MIB2 ubiquitylates multiple sites of cFLIP$_L$. **a** Alignment of C-terminal caspase-like domain of cFLIP$_L$ in various species. Red boxes highlight conserved amino acids among species. Each number indicates the position of the amino acids of human cFLIP$_L$. **b** The dimerization interface of C-terminal domains of cFLIP$_L$ and caspase 8 (adopted from PDB ID codes 3H11) is visualised by RasMol (http://www.openrasmol.org). β6-strand of cFLIP$_L$ is indicated by orange. Red characters indicate four ubiquitylated lysine residues and Q468.

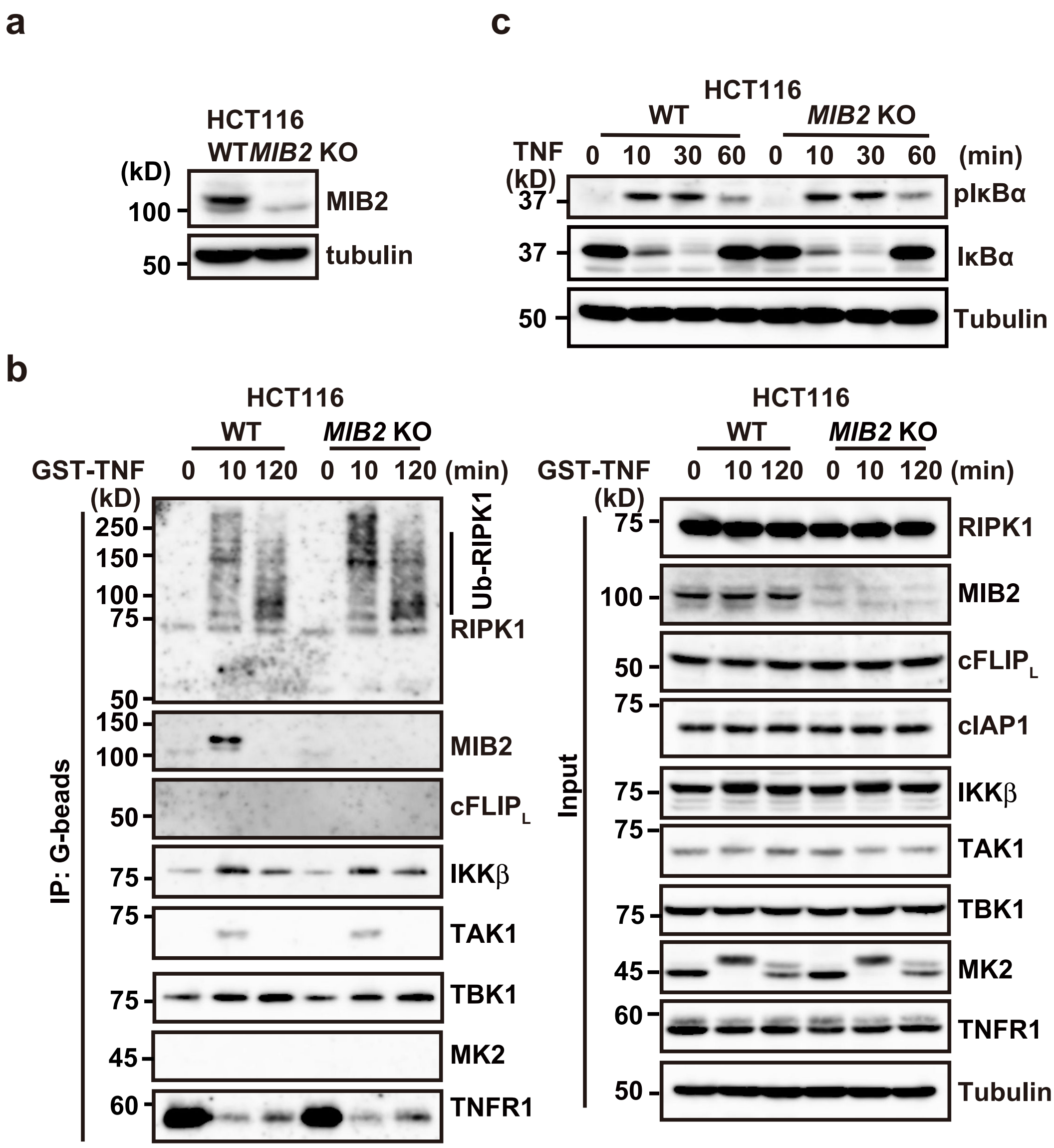

Supplementary Fig. 5 TNF-induces complex I formation is not altered in WT and MIB2 KO HCT 116 cells. a Generation of *MIB2* KO HCT116 cells. Expression of MIB2 was verified by immunoblotting. **b** WT and *MIB2* KO HCT116 cells were stimulated with GST-TNF (1 µg/ml) for the indicated times, and TNFR-containing complex I precipitated with glutathione-Sepharose. Precipitated proteins were analysed by immunoblotting with the indicated antibodies. Protein expression was verified by immunoblotting with the indicated antibodies. **c** WT and *MIB2* KO HCT116 cells were stimulated with TNF for the indicated times, and phosphorylation and degradation of IκBα were analysed by immunoblotting with the indicated antibodies. All results are representative of two or three independent experiments.

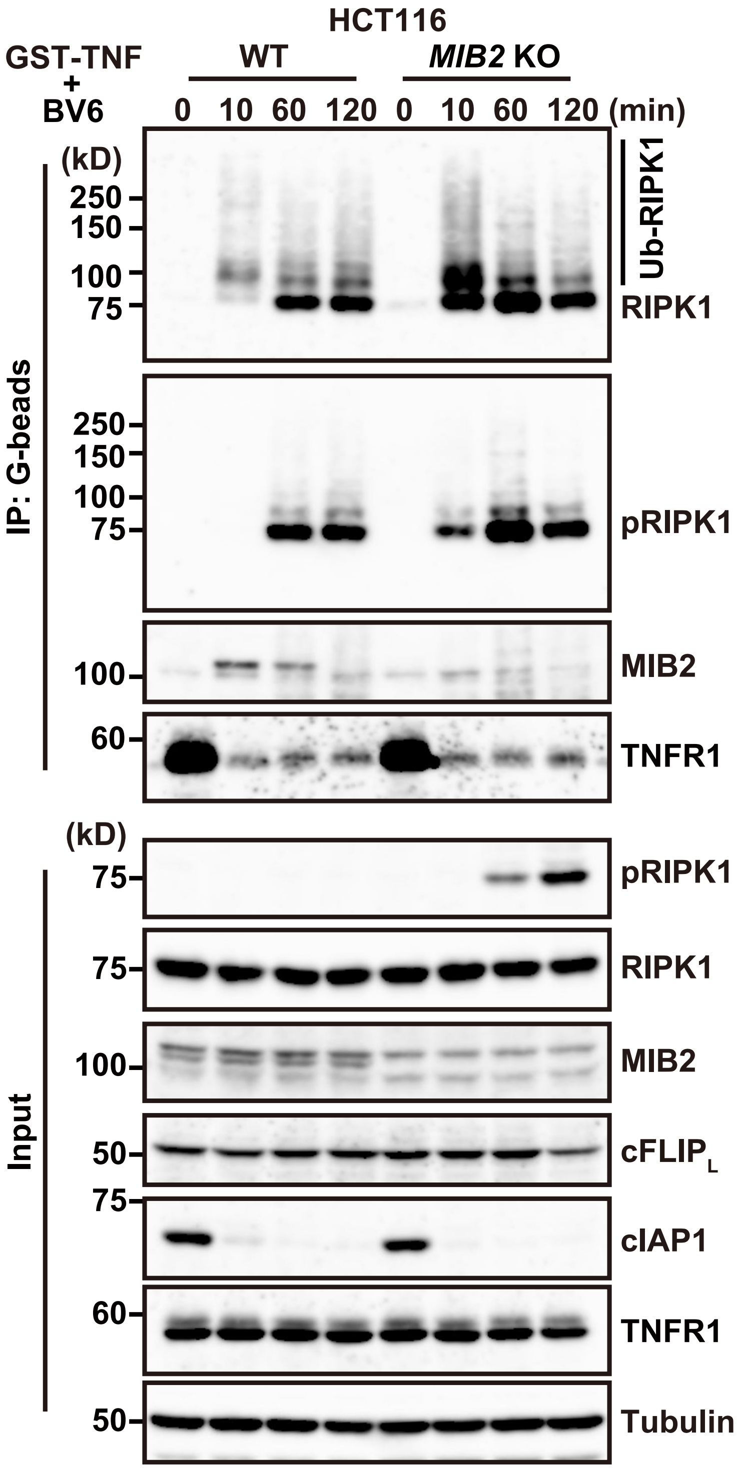

Supplementary Fig. 6 TNF induces phosphorylation of RIPK1 in *MIB2* KO cells in the presence of IAP inhibitor. WT and *MIB2* KO HCT116 cells were stimulated with GST-TNF (1 μg/ml)/BV6 (0.1 μM) for the indicated times, and TNFR-containing complex was precipitated and analysed as in Fig. 6f. Asterisks indicated cross-reacted bands. All results are representative of two or three independent experiments.

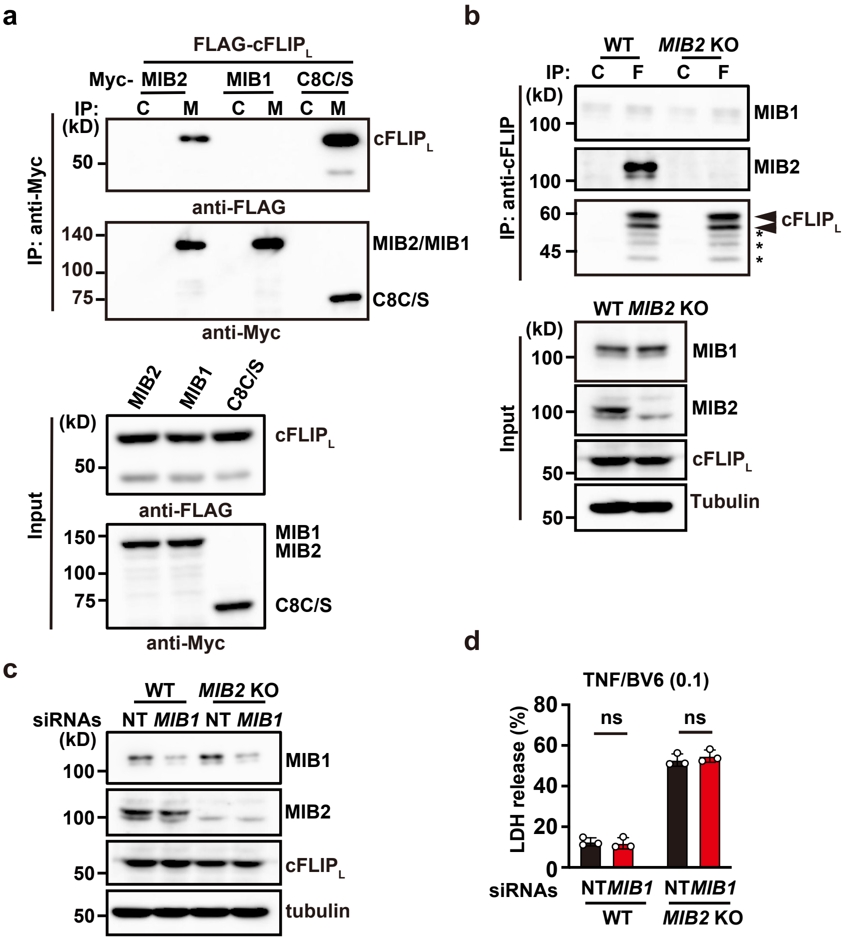

Supplementary Fig. 7 cFLIP$_L$ does not bind to MIB1. a HEK293 cells were transfected with the indicated expression vectors. Cell lysates were immunoprecipitated with control Ig (C) or anti-Myc (M) antibodies, co-immunoprecipitated proteins were analyzed by immunoblotting with anti-FLAG antibody. Protein expression was verified by the indicated antibodies using cell lysates. C8C/S indicates a protease-inactive mutant of caspase 8. **b** WT and *MIB2* KO HeLa cells were immunoprecipitated with control Ig (C) or anti-cFLIP (F) antibody. Immunoprecipitates were analyzed by immunoblotting with anti-MIB1 or anti-MIB2 antibodies. Protein expression was verified by the indicated antibodies. The upper and lower arrowheads indicate modified and unmodified cFLIP$_L$, respectively. Asterisks indicate degraded bands of cFLIP$_L$. **c** WT and *MIB2* KO HeLa cells were transfected with non-target (NT) or *MIB1* siRNAs. Protein expression was verified by immunoblotting with the indicated antibodies. **d** WT and *MIB2* KO HeLa cells were treated with the indicated siRNAs. Sixteen hours after transfection, cells were stimulated with TNF (10 ng/ml) and BV6 (0.1 μM) for 8 hours. Cell death was determined by LDH release assay. Results are mean ± SD of triplicate samples. Unpaired two-tailed Student *t*-test. ns, not significant. All results are representative of two independent experiments.

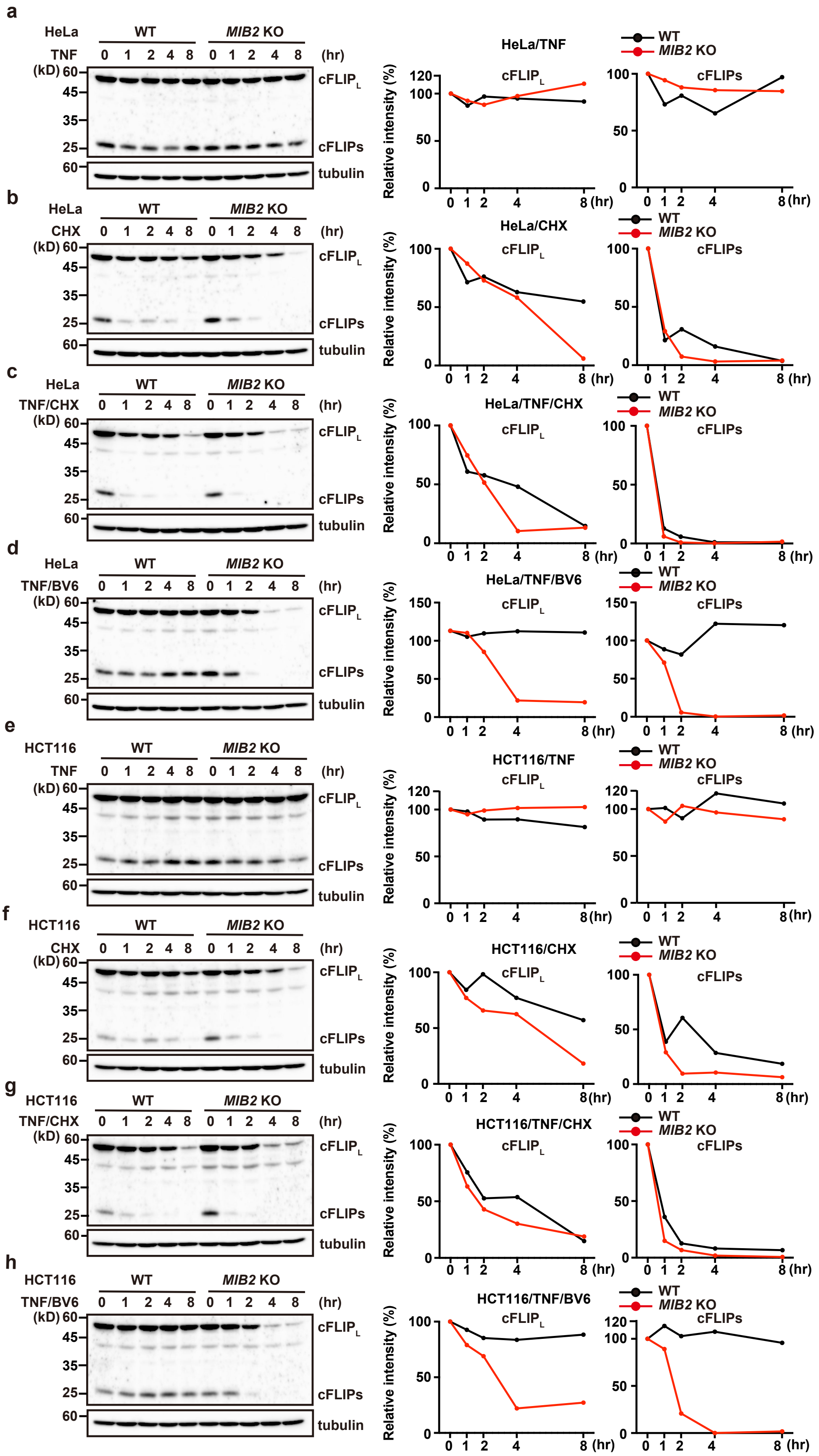
a
HeLa
WT
MIB2 KO
TNF
0 1 2 4 8 0 1 2 4 8 (hr)
(kD) 60
45
35
25
60
cFLIPL
cFLIPs
tubulin
HeLa/TNF
Relative intensity (%)
WT
MIB2 KO
b
HeLa
CHX
HeLa/CHX
c
HeLa
TNF/CHX
HeLa/TNF/CHX
d
HeLa
TNF/BV6
HeLa/TNF/BV6
e
HCT116
TNF
HCT116/TNF
f
HCT116
CHX
HCT116/CHX
g
HCT116
TNF/CHX
HCT116/TNF/CHX
h
HCT116
TNF/BV6
HCT116/TNF/BV6

Supplementary Fig. 8 MIB2 does not promote degradation but rather increases the stability of cFLIP$_L$. a-d WT and *MIB2* KO HeLa were stimulated with TNF (1 ng/ml) (**a**), CHX (2.5 μg/ml) (**b**), TNF (1 ng/ml)/CHX (2.5 μg/ml) (**c**), or TNF (10 ng/ml)/BV6 (0.1 μM) (**d**) for the indicated times, and expression of cFLIP$_L$ and cFLIPs was analyzed by immunoblotting with anti-cFLIP antibody. Signal intensities of cFLIP$_L$, cFLIPs, and tubulin at the indicated times were calculated by Image J and normalized by those of tubulin. Relative intensities of cFLIP$_L$ and cFLIPs at the indicated times compared to those at time 0 (100%) are plotted. **e-h** WT and *MIB2* KO HCT116 were stimulated with TNF (1 ng/ml) (**e**), CHX (2.5 μg/ml) (**f**), TNF (1 ng/ml)/CHX (2.5 μg/ml) (**g**), or TNF (10 ng/ml)/BV6 (0.1 μM) (**h**) for the indicated times, and analyzed as in (**a**). Results are representative of two independent experiments.

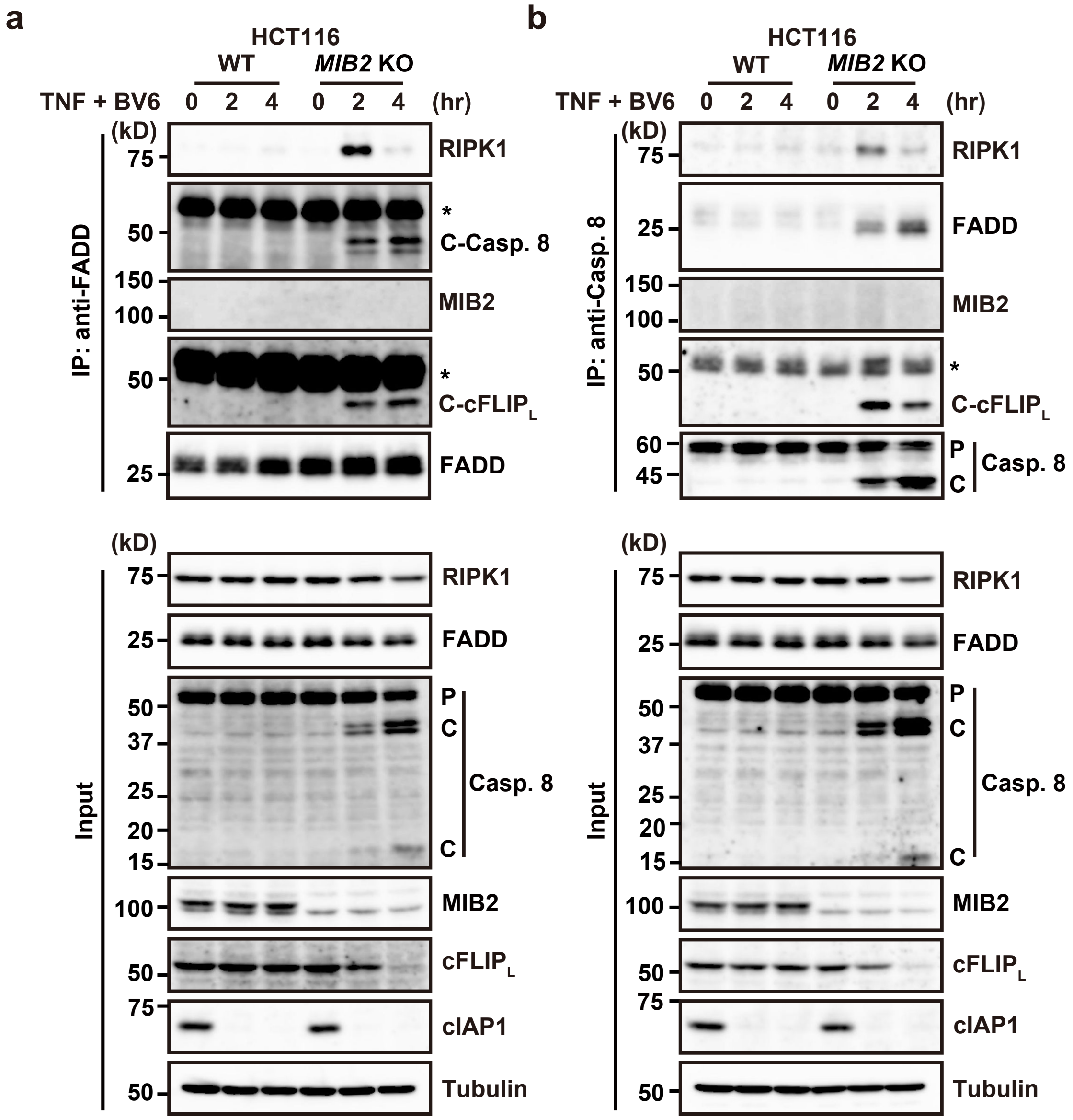

Supplementary Fig. 9 Complex IIb formation is facilitated in *MIB2* KO HCT116 cells. a, **b** WT and *MIB2* KO HCT116 cells were stimulated with TNF (10 ng/ml) and BV6 (0.1 μM) for the indicated times, and cell lysates were immunoprecipitated with anti-FADD (**a**) or anti-caspase 8 (**b**) antibodies. Immunoprecipitated proteins were analyzed by immunoblotting with the indicated antibodies. Protein expression was verified by immunoblotting with the indicated antibody. *Cross-reacted band; C-Casp. 8, cleaved caspase 8; C-cFLIP$_L$, cleaved cFLIP$_L$. P and C indicate proform and cleaved form, respectively. All results are representative of two or three independent experiments.

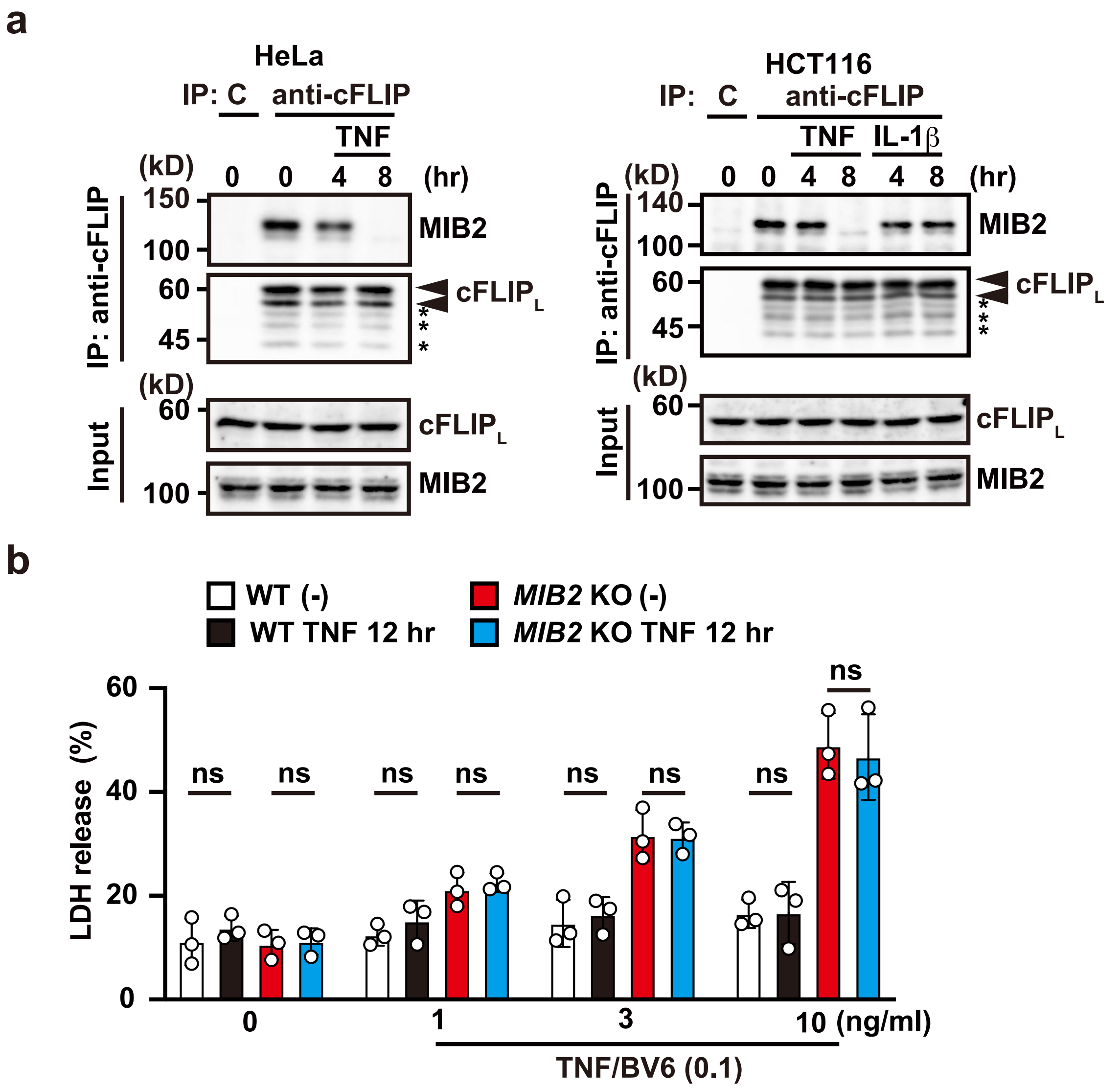

Supplementary Fig. 10 TNF induces dissociation of the complex of MIB2 and $cFLIP_L$. a HeLa or HCT116 cells were stimulated with TNF (10 ng/ml) or IL-1β (10 ng/ml) for the indicated times, and cell lysates immunoprecipitated with anti-cFLIP antibody. Immunoprecipitated MIB2 and cFLIPL were analysed by immunoblotting with anti-MIB2 and anti-cFLIP antibodies, respectively. The upper and lower arrowheads indicate modified and unmodified $cFLIP_L$, respectively. Asterisks indicate degraded bands of $cFLIP_L$. Protein expression was verified by immunoblotting with the indicated antibody. **b** WT and *MIB2* KO HeLa cells were pretreated or not with TNF (10 ng/ml) for 12 hours and then stimulated with the indicated concentrations of TNF (ng/ml) and BV6 (0.1 μM) for 8 hours. Cell death was determined by the LDH release assay. Results are mean ± SD of triplicate samples. Unpaired two-tailed Student *t*-test. ns, not significant.

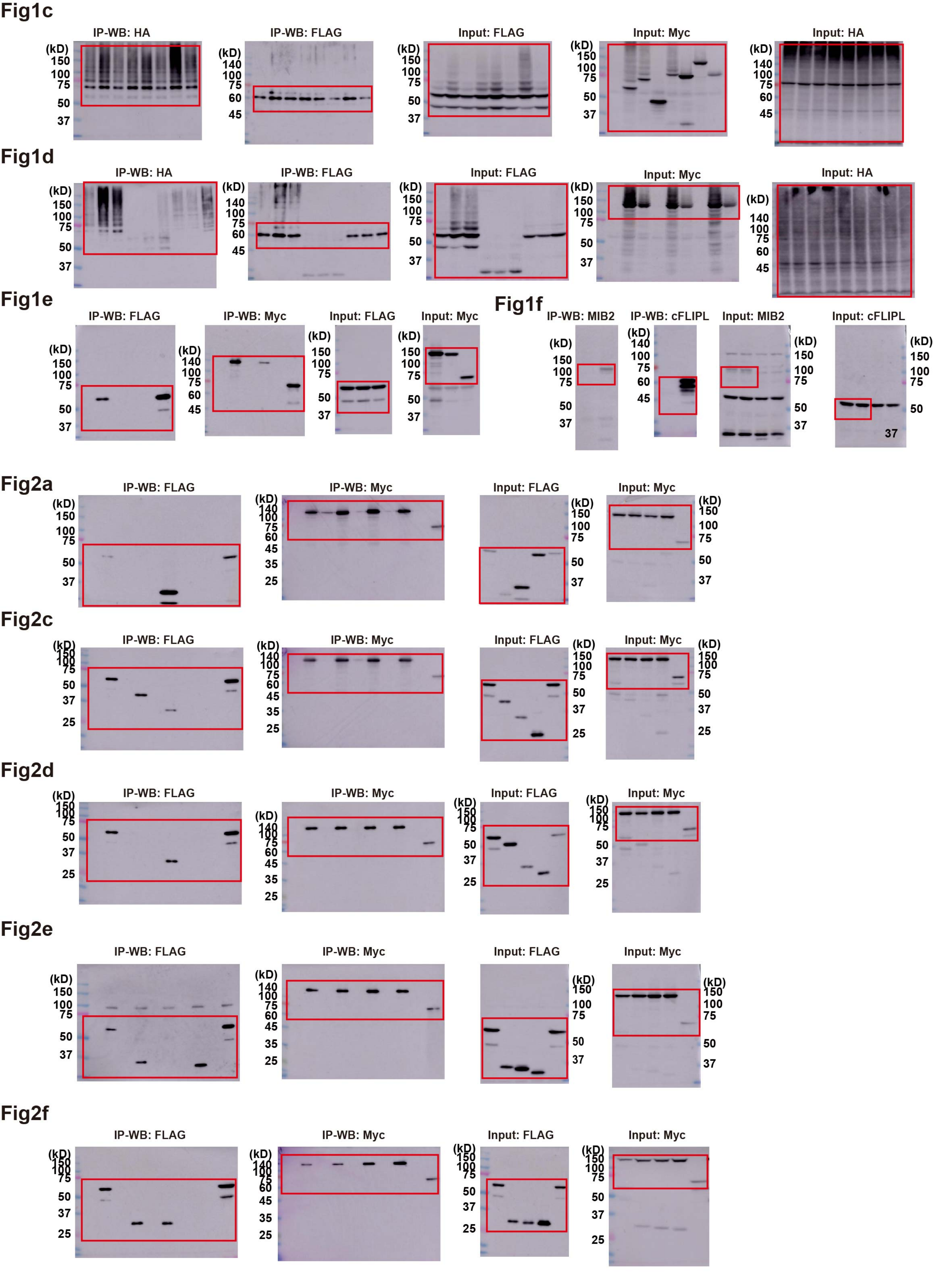

Supplementary Figure 11 | Uncropped blots for Figs. 1 and 2.

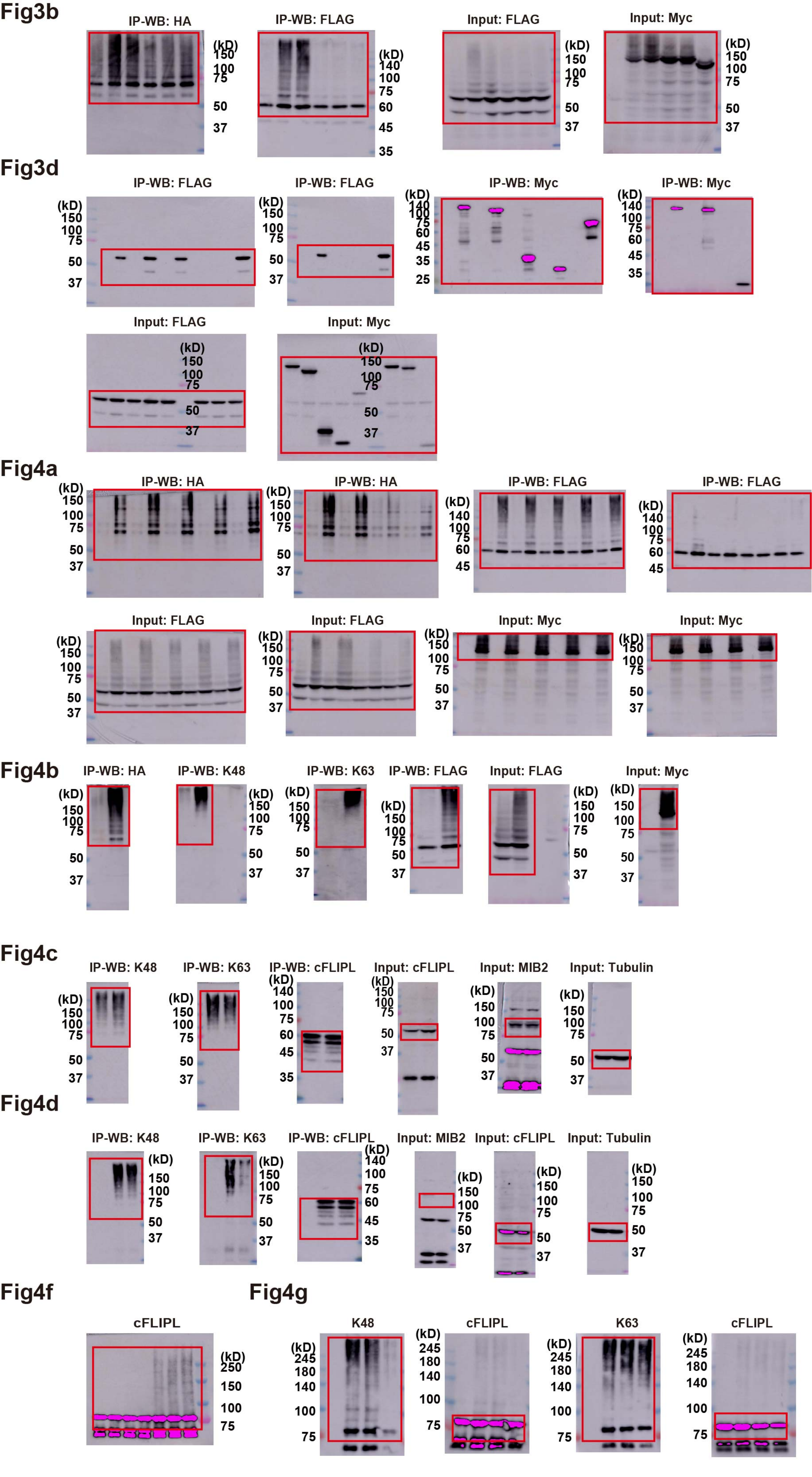

Supplementary Figure 12 | Uncropped blots for Figs. 3 and 4.

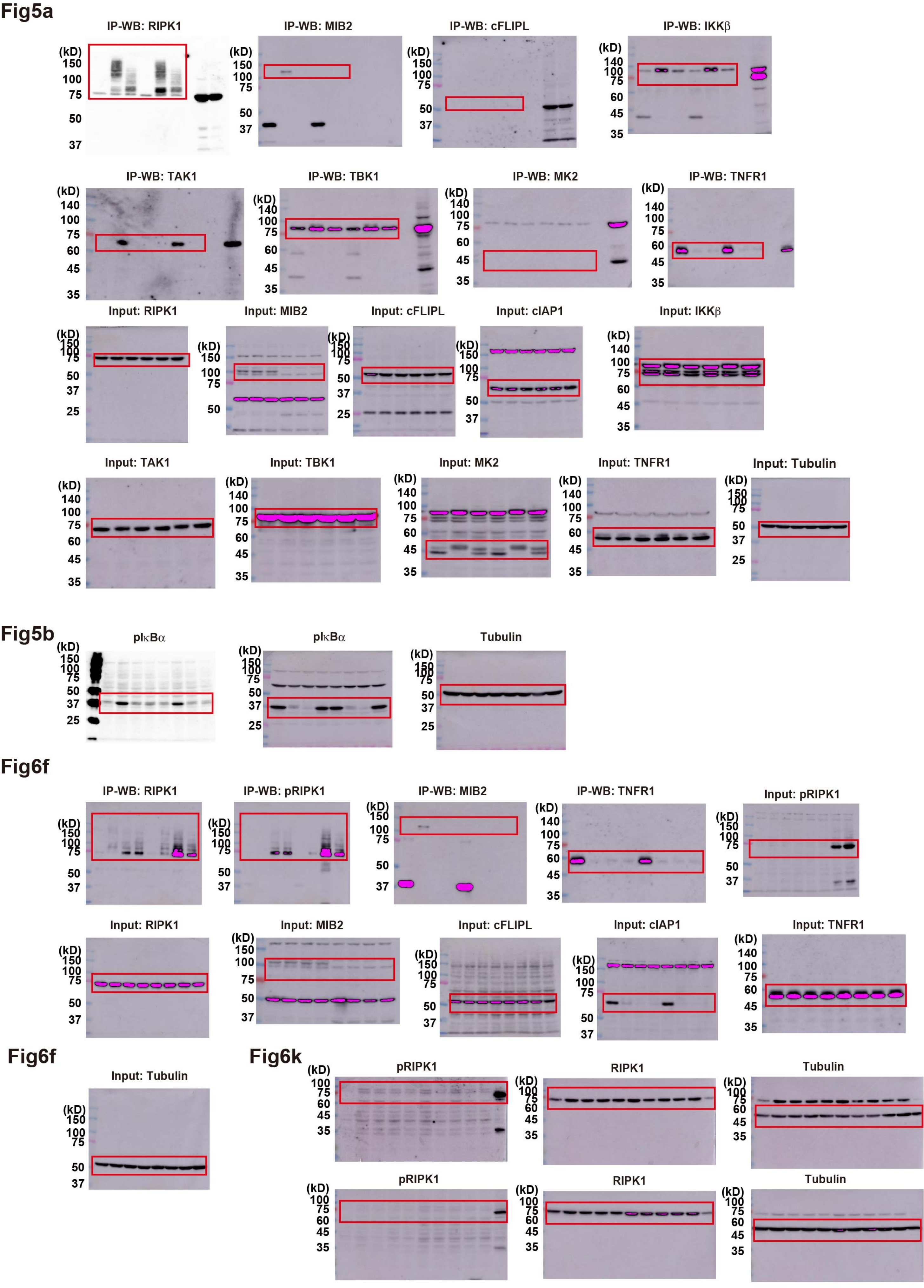

Supplementary Figure 13 | Uncropped blots for Figs. 5 and 6.

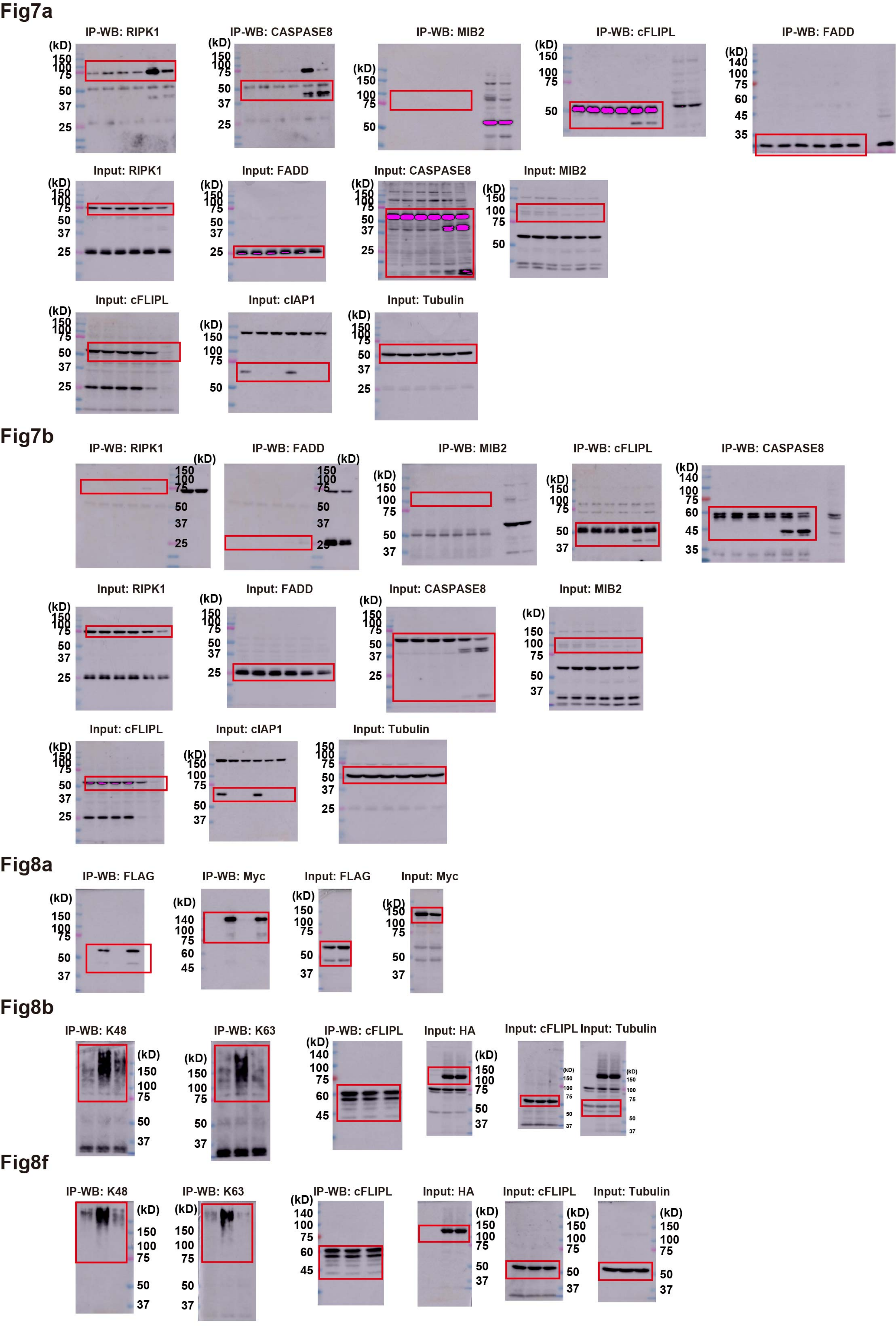

Supplementary Figure 14 | Uncropped blots for Figs. 7 and 8.

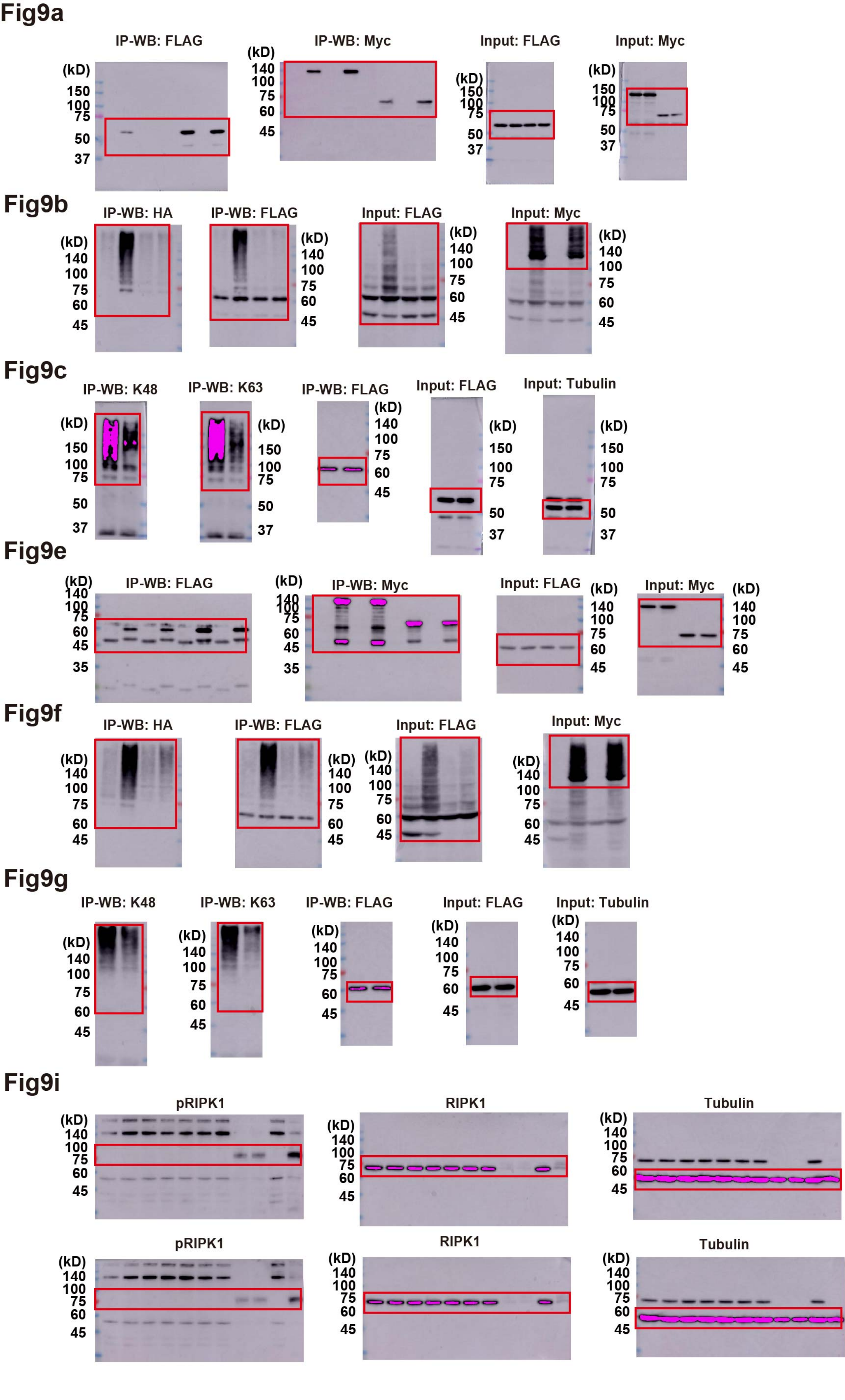

Supplementary Figure 15 | Uncropped blots for Fig. 9.

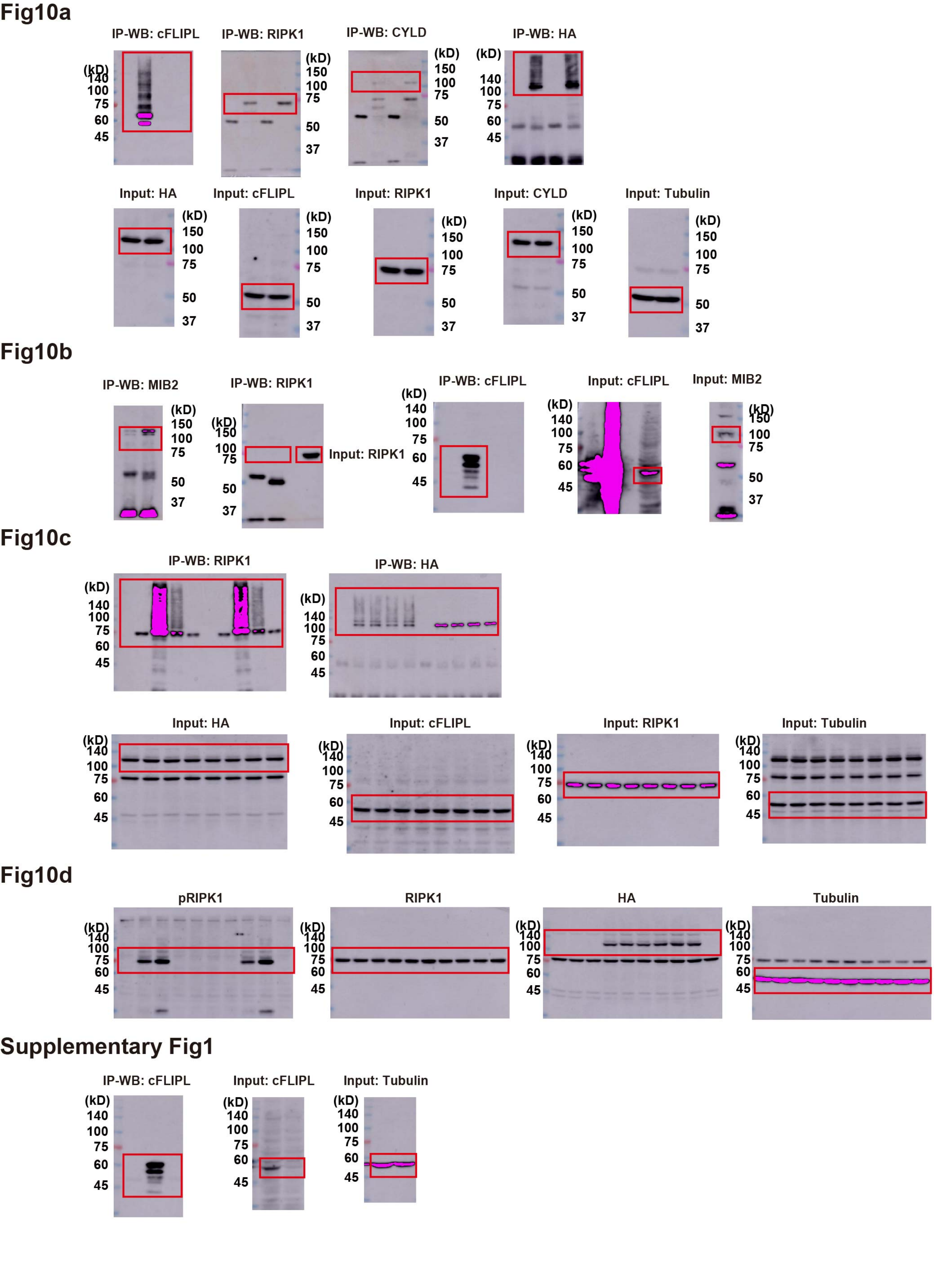

Supplementary Figure 16 | Uncropped blots for Fig. 10 and Supplementary Fig. 1.

Supplementary Fig3a

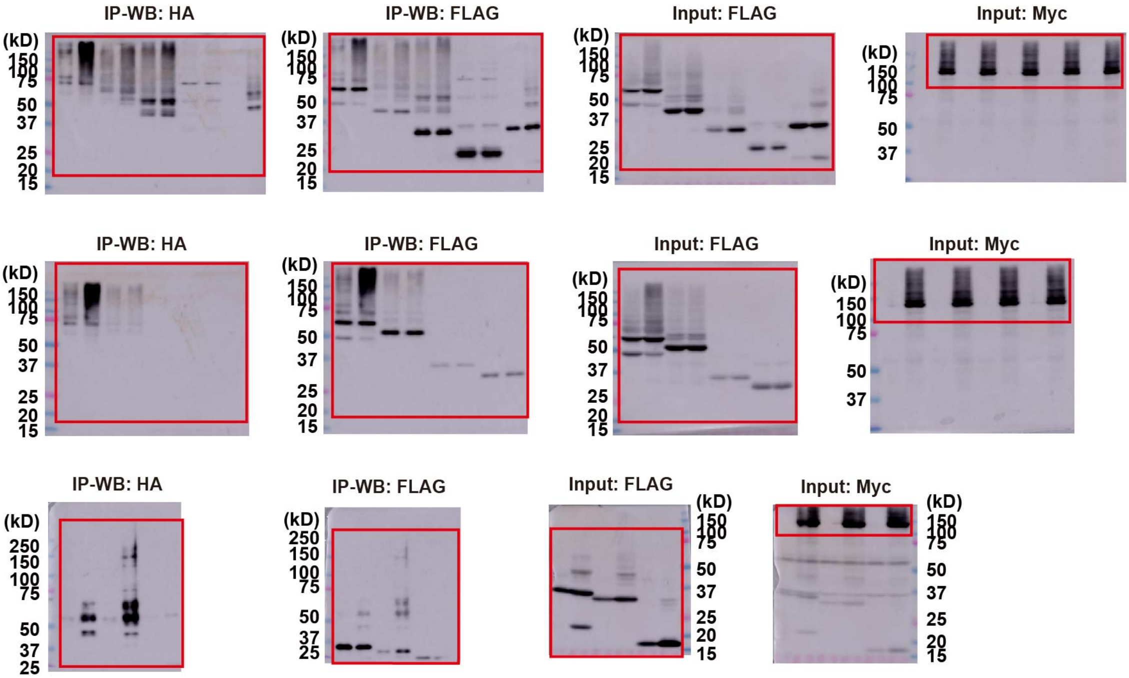

Supplementary Fig3c

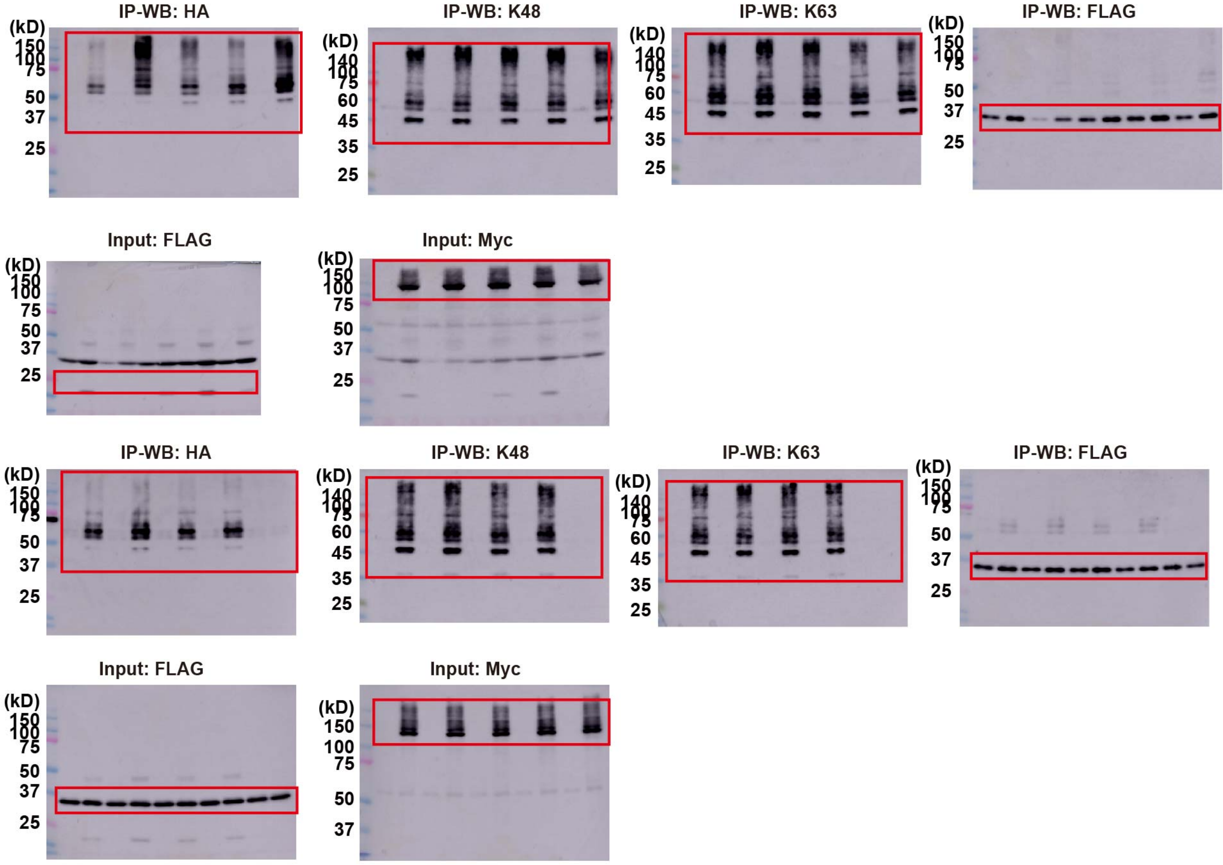

Supplementary Figure 17 | Uncropped blots for Supplementary Fig. 3.

Supplementary Fig5a

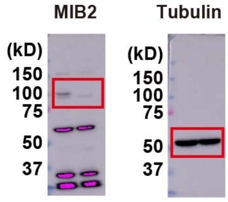

Supplementary Fig5b

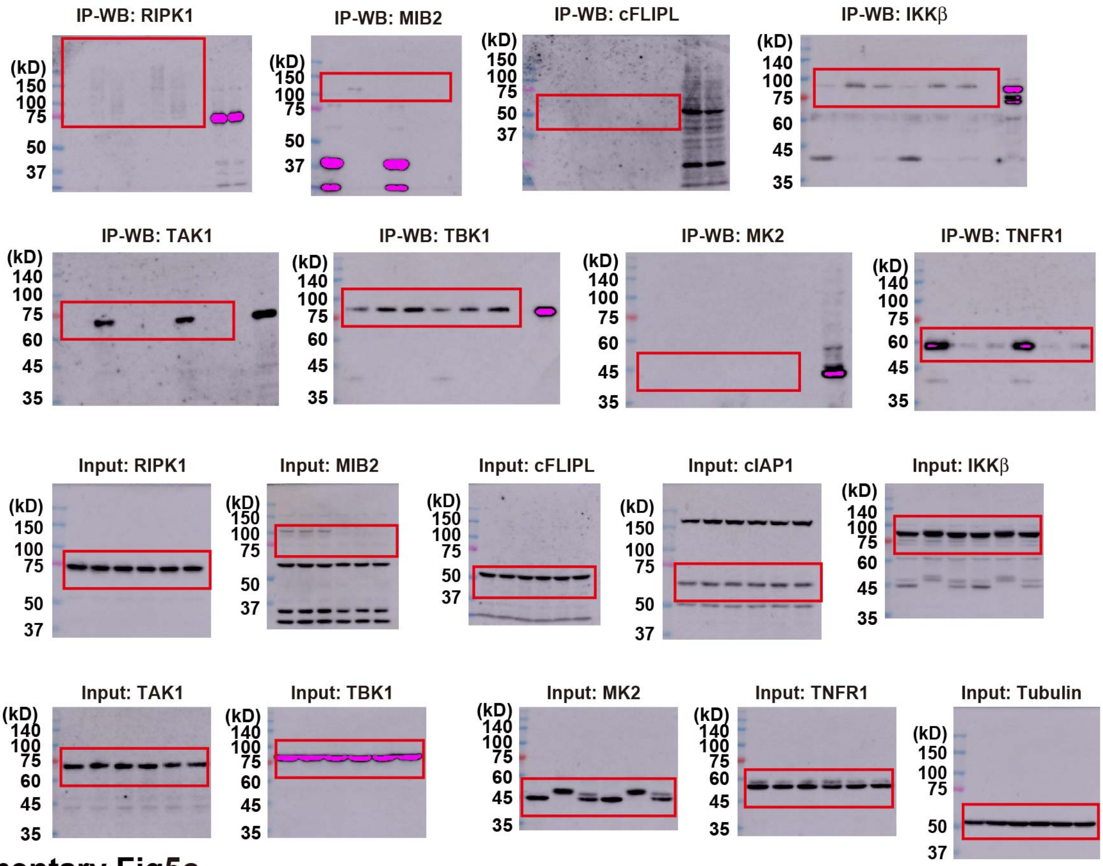

Supplementary Fig5c

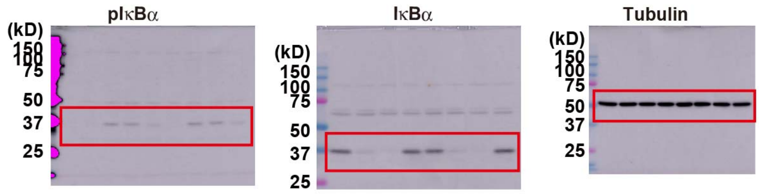

Supplementary Fig6

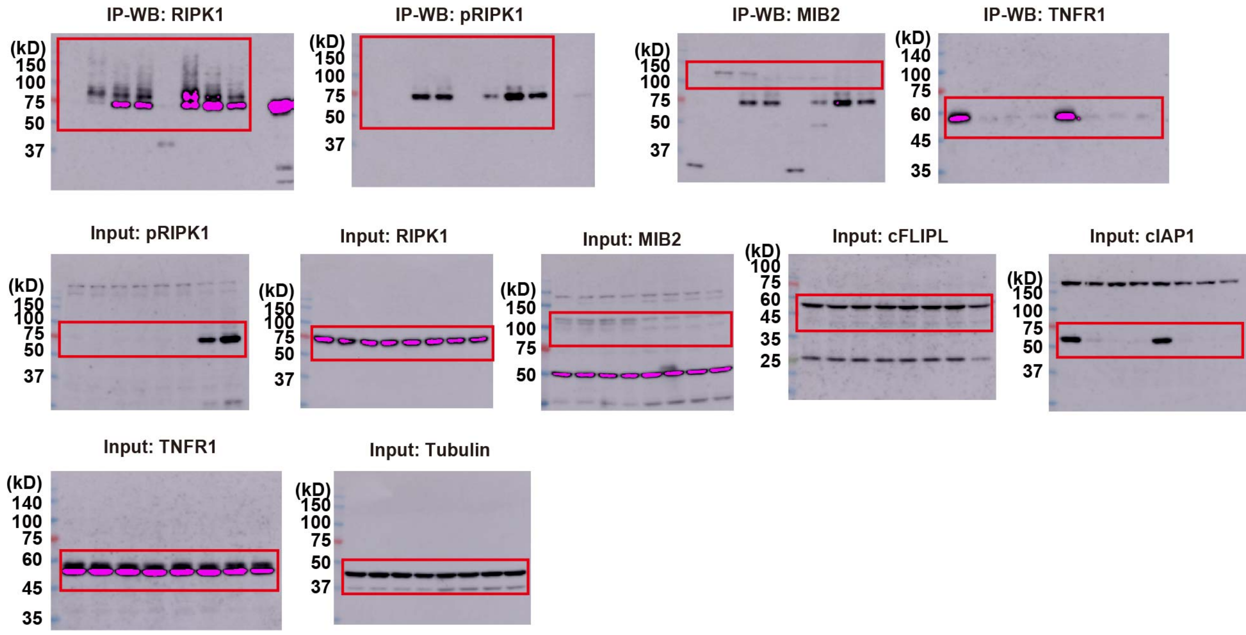

Supplementary Figure 18 | Uncropped blots for Supplementary Figs. 5 and 6.

Supplementary Fig7a

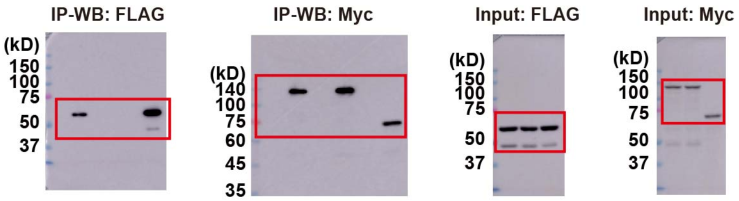

Supplementary Fig7b

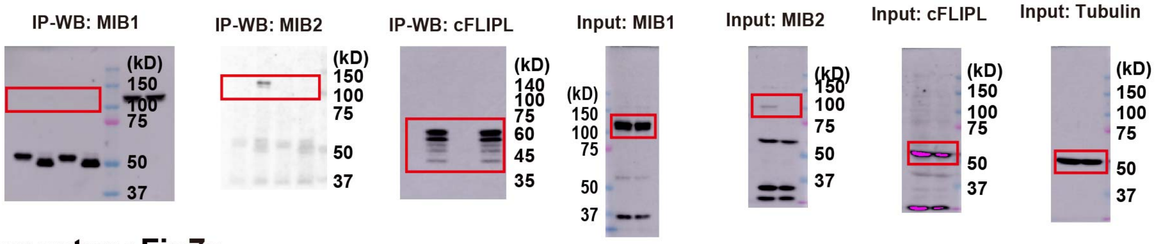

Supplementary Fig7c

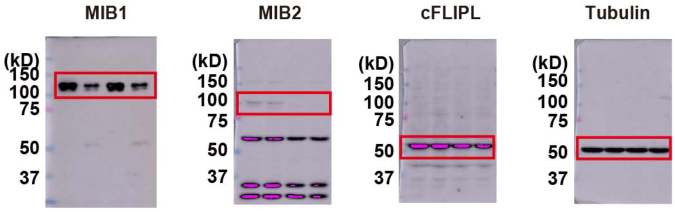

Supplementary Fig8a-h

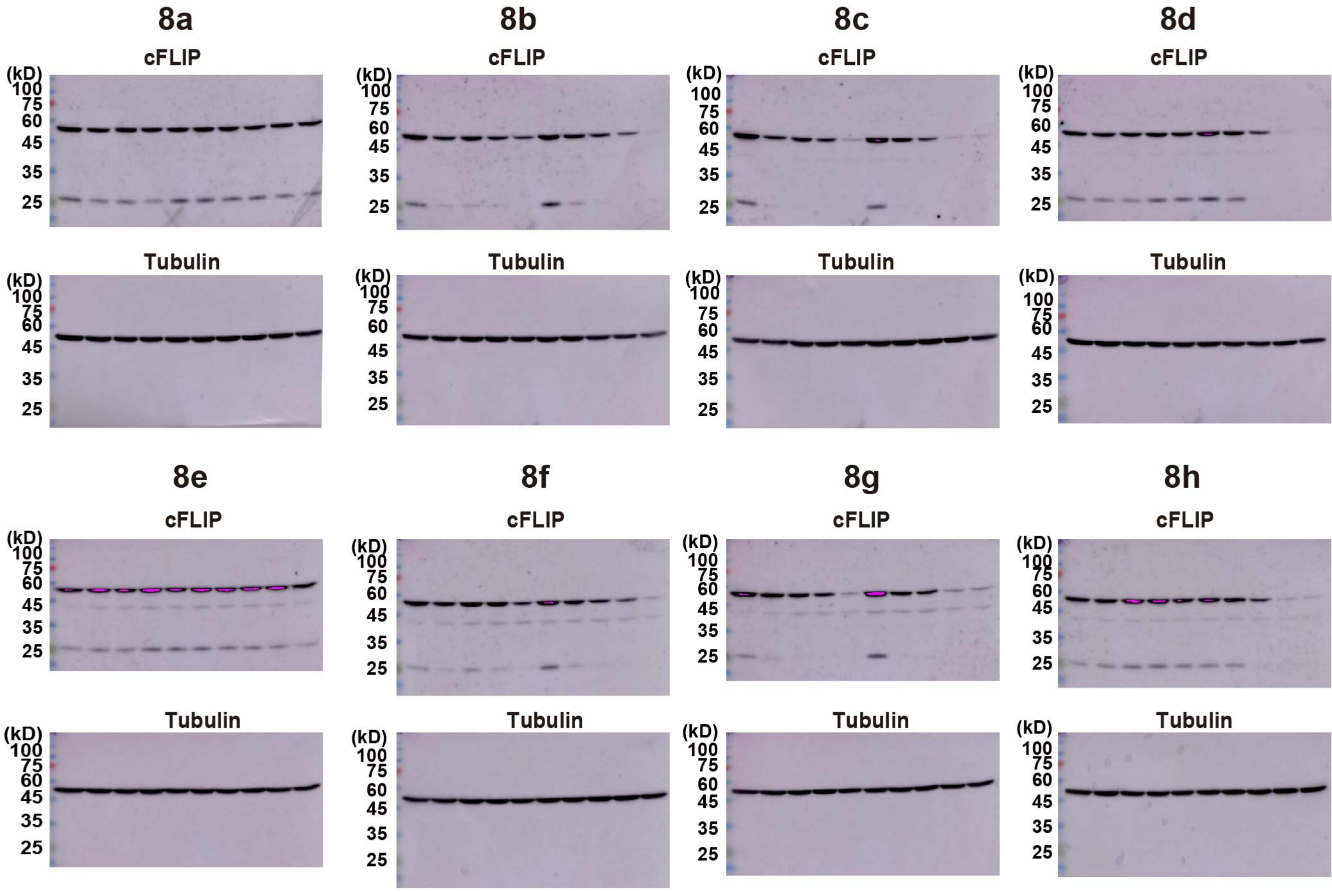

Supplementary Figure 19 | Uncropped blots for Supplementary Figs. 7 and 8.

Supplementary Fig9a

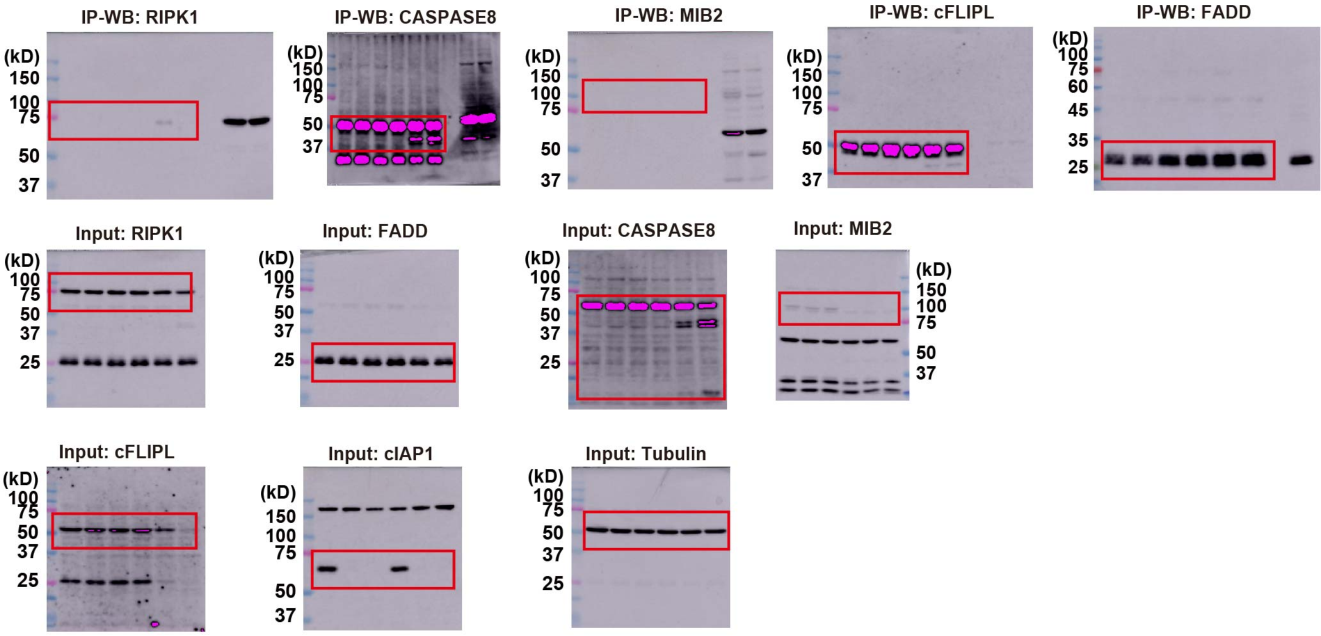

Supplementary Fig9b

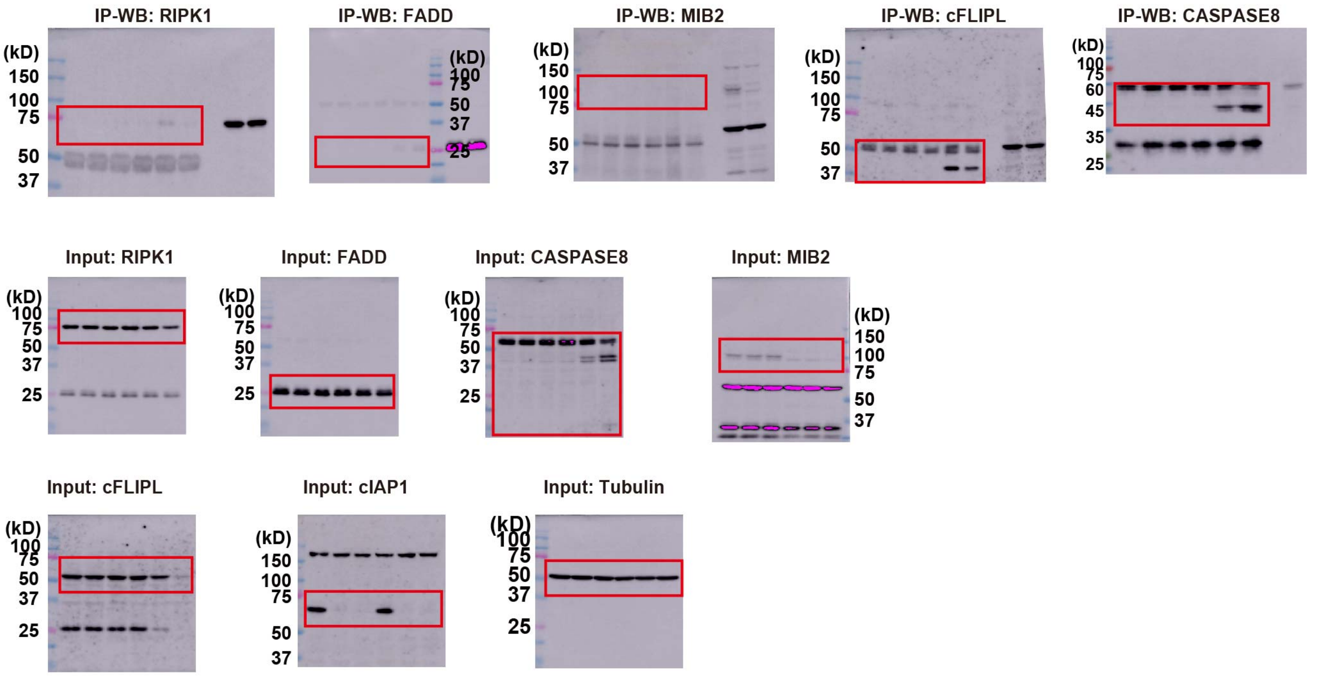

Supplementary Fig10a

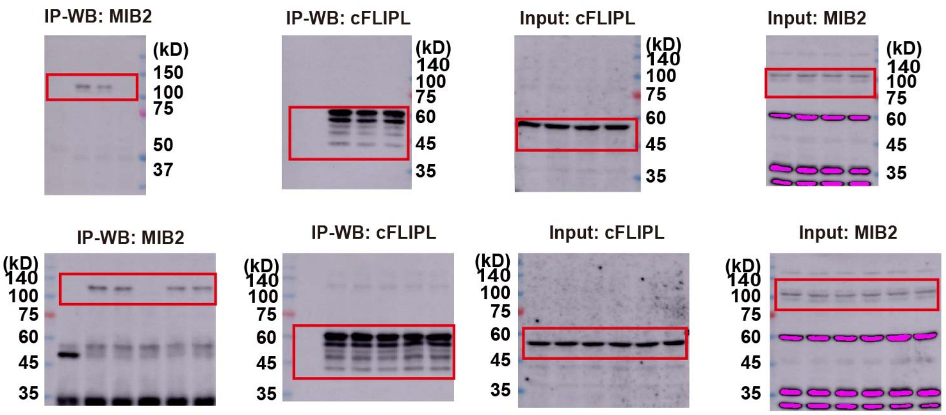

Supplementary Figure 20 | Uncropped blots for Supplementary Figs. 9 and 10.
