# Supplementary material for: MIND bomb 2 prevents RIPK1 kinase activity-dependent and -independent apoptosis through ubiquitylation of cFLIPL
Source: Commun Biol. 2021 Jan 19;4:80. doi: 10.1038/s42003-020-01603-y (PMC7815719; doi:10.1038/s42003-020-01603-y)
Supplement: Supplementary file 3 — Description of Additional Supplementary Files [file 42003_2020_1603_MOESM3_ESM.pdf]

**Description of Additional Supplementary Files**

File Name: Supplementary Data 1
Description: Binding scores of E3 ligases to cFLIPL.

File Name: Supplementary Data 2
Description: Statistical analysis of the results in Fig. 4.

File Name: Supplementary Data 3
Description: Statistical analysis of the results in Fig. 6.

File Name: Supplementary Data 4
Description: Statistical analysis of the results in Fig. 8.

File Name: Supplementary Data 5
Description: Statistical analysis of the results in Fig. 9.

File Name: Supplementary Data 6
Description: Statistical analysis of the results in Supplementary Fig. 7.

File Name: Supplementary Data 7
Description: Statistical analysis of the results in Supplementary Fig. 8.

File Name: Supplementary Data 8
Description: Statistical analysis of the results in Supplementary Fig. 10.
