# Supplementary material for: MIND bomb 2 prevents RIPK1 kinase activity-dependent and -independent apoptosis through ubiquitylation of cFLIPL
Source: Commun Biol. 2021 Jan 19;4:80. doi: 10.1038/s42003-020-01603-y (PMC7815719; doi:10.1038/s42003-020-01603-y)
Supplement: Supplementary file 12 — Reporting Summary [file 42003_2020_1603_MOESM12_ESM.pdf]

nature research

Corresponding author(s): Hiroyasu Nakano
Last updated by author(s): Nov 26, 2020

Reporting Summary

Nature Research wishes to improve the reproducibility of the work that we publish. This form provides structure for consistency and transparency in reporting. For further information on Nature Research policies, see our Editorial Policies and the Editorial Policy Checklist.

Statistics

For all statistical analyses, confirm that the following items are present in the figure legend, table legend, main text, or Methods section.

n/a	Confirmed	
☐	☒	The exact sample size (n) for each experimental group/condition, given as a discrete number and unit of measurement
☐	☒	A statement on whether measurements were taken from distinct samples or whether the same sample was measured repeatedly
☐	☒	The statistical test(s) used AND whether they are one- or two-sided<br>*Only common tests should be described solely by name; describe more complex techniques in the Methods section.*
☒	☐	A description of all covariates tested
☒	☐	A description of any assumptions or corrections, such as tests of normality and adjustment for multiple comparisons
☐	☒	A full description of the statistical parameters including central tendency (e.g. means) or other basic estimates (e.g. regression coefficient) AND variation (e.g. standard deviation) or associated estimates of uncertainty (e.g. confidence intervals)
☐	☒	For null hypothesis testing, the test statistic (e.g. F, t, r) with confidence intervals, effect sizes, degrees of freedom and P value noted<br>*Give P values as exact values whenever suitable.*
☒	☐	For Bayesian analysis, information on the choice of priors and Markov chain Monte Carlo settings
☒	☐	For hierarchical and complex designs, identification of the appropriate level for tests and full reporting of outcomes
☒	☐	Estimates of effect sizes (e.g. Cohen's d, Pearson's r), indicating how they were calculated

Our web collection on statistics for biologists contains articles on many of the points above.

Software and code

Policy information about availability of computer code

Data collection	SoftWoRx software (version 7.0)
Data analysis	GraphPad_Prism (version 8.0); SoftWoRx software (version 7.0); Image J; Adobe Illustrator 2020; Adobe Photoshop Element 6

For manuscripts utilizing custom algorithms or software that are central to the research but not yet described in published literature, software must be made available to editors and reviewers. We strongly encourage code deposition in a community repository (e.g. GitHub). See the Nature Research guidelines for submitting code & software for further information.

Data

Policy information about availability of data

All manuscripts must include a data availability statement. This statement should provide the following information, where applicable:
- Accession codes, unique identifiers, or web links for publicly available datasets
- A list of figures that have associated raw data
- A description of any restrictions on data availability

The authors declare that the data supporting the findings of this study are available within the paper and its supplementary information files. Data not included are available from the corresponding authors upon reasonable request.

Field-specific reporting

Please select the one below that is the best fit for your research. If you are not sure, read the appropriate sections before making your selection.

☒ Life sciences ☐ Behavioural & social sciences ☐ Ecological, evolutionary & environmental sciences

For a reference copy of the document with all sections, see nature.com/documents/nr-reporting-summary-flat.pdf

Life sciences study design

All studies must disclose on these points even when the disclosure is negative.

Sample size	For LDH release assays, we used triplicate samples. For immunofluorescent analysis, at least 5 cell images were collected per each transfection. We performed all experiments at least twice to confirm reproducibility of the results.
Data exclusions	No.
Replication	Each experiments was repeated at least twice or three times.
Randomization	No.
Blinding	No.

Reporting for specific materials, systems and methods

We require information from authors about some types of materials, experimental systems and methods used in many studies. Here, indicate whether each material, system or method listed is relevant to your study. If you are not sure if a list item applies to your research, read the appropriate section before selecting a response.

Materials & experimental systems

n/a	Involved in the study	
☐	☒	Antibodies
☐	☒	Eukaryotic cell lines
☒	☐	Palaeontology and archaeology
☒	☐	Animals and other organisms
☒	☐	Human research participants
☒	☐	Clinical data
☒	☐	Dual use research of concern

Methods

n/a	Involved in the study	
☒	☐	ChIP-seq
☒	☐	Flow cytometry
☒	☐	MRI-based neuroimaging

Antibodies

Antibodies used

mouse monoclonal anti-FLAG (M2, Sigma-Aldrich, 1:1000),
mouse monoclonal anti-HA (12CA5, Sigma-Aldrich, 1:1000),
rat monoclonal anti-HA (3F10, Sigma-Aldrich, 1:1000),
mouse monoclonal anti-Myc (9E10, Sigma-Aldrich, 1:1000),
mouse monoclonal anti-caspase 8 (9746, 1C12, Cell Signaling Technology, 1:1000)
rabbit anti-caspase 8 (13423-1-AP, Proteintech, for IP)
mouse monoclonal anti-cFLIP (7F10, Enzo, 1:1000),
rat monoclonal anti-cFLIP (Dave-2, Adipogen, 1:1000)
gout anti-cylindromatosis (21286, Santa Cruz, 1:1000)
mouse monoclonal anti-FADD (056-486, 1F7, Sigma-Aldrich, 1:1000),
rabbit anti-FADD (14906-1-1-AP, Proteintech, for IP),
rabbit anti-IkappaBalpha(371, C-21, Santa Cruz, 1:1000),
rabbit monoclonal anti-phospho-IkappaBalpha(2859, 14D4, Cell Signaling Technology, 1:1000),
rabbit anti-human MIB1 (393551, D-6, Santa Cruz, 1:1000),
rabbit anti-human MIB2 (ab99378, Abcam, 1:1000),
anti-MK2 (3042, Cell Signaling Technology, 1:1000),
rat monoclonal anti-cIAP1 (1E1-1-10, Enzo, 1:1000),
rabbit monoclonal anti-K48 (05-1307, Apu2, Sigma-Aldrich, 1:1000),
rabbit monoclonal anti-K63 (ab179434, Abcam, 1:1000),
rabbit monoclonal anti-phospho-RIPK1 (31122, Cell Signaling Technology, 1:1000)
mouse monoclonal anti-RIPK1 (610459, BD Transduction Lab, 1:1000)
rabbit monoclonal anti-TAK1 (5206, Cell Signaling Technology, 1:1000),
rabbit monoclonal anti-TBK1 (3504, Cell Signaling Technology, 1:1000),
mouse monoclonal anti-TNFR1 (8436, Santa Cruz, 1:1000),

	mouse monoclonal anti-alpha-tubulin (T5168, Sigma-Aldrich, 40,000)<br>Secondary Antibodies:<br>HRP–conjugated donkey anti-rabbit IgG (NA934, GE Healthcare Life Science, 1:10000)<br>HRP-conjugated goat anti-rat IgG (NA935, GE Healthcare Life Science, 1:10000)<br>HRP-conjugated sheep anti-mouse IgG (NA931, GE Healthcare Life Science, 1:10000)
Validation	All antibodies were validated by each company. For some antibodies used for the first time in this study, we checked the specificity of these antibodies using cells transfected with respective siRNAs or knockout cells.

Eukaryotic cell lines

Policy information about cell lines

Cell line source(s)	HeLa, HCT116, HEK293 cells were derived from ATCC. Cflar-/- murine embryonic fibroblasts were characterized in the paper in Immunity (Yeh et al, 2000) and obtained from Wen-Chen Yeh.
Authentication	All cell lines were previously published and well characterized by many investigators.
Mycoplasma contamination	Not determined.
Commonly misidentified lines<br>(See ICLAC register)	Not applicable.
